# Cystic plate approach combined with ICG fluorescence in laparoscopic anatomical hepatectomy

**DOI:** 10.1097/JS9.0000000000001706

**Published:** 2024-05-29

**Authors:** Hao Chen, Kefeng Shen, Xiayong Shen, Wenbin Liu, Yongsheng Ge, Jihai Yu, Weidong Jia, Jinliang Ma, Peng Yuan, Chuanhai Zhang

**Affiliations:** a Department of Hepatic Surgery, The First Affiliated Hospital of USTC, Division of Life Sciences and Medicine, University of Science and Technology of China, HeFei; b Department of Graduate School, Wannan Medical College, Wuhu, People’s Republic of China

**Keywords:** anatomic, cystic plate, hepatectomy, indocyanine green, laparoscopy

## Abstract

**Background:**

The in-depth understanding of the fine anatomy of the liver has promoted the development of modern liver surgery. With the rapid popularity of laparoscopic hepatectomy, the membrane structure of the liver and its ability to dissect the intrahepatic and extrahepatic vascular system more conveniently and accurately has been gradually emphasized.

**Objective:**

Exploring the value of extrahepatic sheath dissection of the hepatic pedicle in minimally invasive anatomical hepatectomy with cystic plate approach. This study aims to assess the benefits of integrating the cystic plate approach with real-time guided laparoscopic anatomical hepatectomy, in comparison with conventional laparoscopic anatomical hepatectomy.

**Materials and methods:**

Based on the theory of cystic plate and hepatic portal plate, the authors have pioneered the fluorescence real-time guided cystic plate approach in hepatectomy. The article focuses on the anatomical knowledge and technical difficulties of anatomical hepatectomy with fluoroscopic laparoscopic cystic plate approach and explores the safety and practicality of the cystic plate approach in laparoscopic anatomical hepatectomy. Additionally, a retrospective cohort study was also conducted to compare the operation time, intraoperative blood loss, and postoperative complications between the cystic plate approach and the conventional approach during fluoroscopic laparoscopic hepatectomy.

**Results:**

A total of 38 patients who met the inclusion criteria underwent laparoscopic hepatectomy between January 2019 and November 2022. No significant disadvantages were found in terms of operation time and intraoperative blood loss during the surgeries. Furthermore, the postoperative indications, including liver function indexes on the first postoperative day, WBC, and the postoperative hospital stay, were also not affected, thus proving the safety of the cystic approach. Importantly, through the cystic plate approach, the target liver pedicle was fully freed, and then the segments to be resected were precisely marked by positive or negative staining, followed by hepatectomy under real-time fluoroscopic guidance. This approach is extremely advantageous in anatomical liver segment resections, especially in right posterior lobe or hemi-hepatectomy, without increasing intraoperative bleeding or postoperative complication rates.

**Conclusion:**

This technique allows for easy and safe freeing of the target liver pedicle using membrane structures, and also allows for precise anatomical hepatectomy in combination with real-time fluoroscopic laparoscopic navigation.

Highlights

We redefining the theory and approach to the Cystic plate.

- Redefined the Cystic plate approach from the membrane anatomy theory, and unlike the previous approach that started from the gallbladder triangle.
- Innovated the approach from the ‘red/yellow junction line’ at the bottom of the gallbladder.
- Easily and safety freeing the target hepatic hilum by utilizing the membrane structure and precise anatomical hepatectomy by combining the real-time navigation of fluorescent laparoscopy.

Liver cancer is one of the most common malignant tumors of the digestive system, with the sixth highest incidence rate and the second highest mortality rate worldwide. Surgical resection is still the preferred treatment for liver cancer^1^. With the introduction of membrane anatomy theory, laparoscopic hepatectomy has entered a new era^2^. The separation of the cystic plate begins with cholecystectomy, from the base of the gallbladder to the neck and the liver parenchyma, then the right anterior, right posterior, and left hepatic pedicle can be easily and quickly revealed by freeing to the hepatic portal plate, which called ‘the cystic plate approach to hepatectomy’^3^. After isolation of the hepatic pedicle, indocyanine green (ICG) fluorescence staining is feasible, and intraoperative navigation of the resection plane is performed in real-time^4^; even in the presence of cirrhosis, ultrasonic knife can well control bleeding from the cystic plate and liver parenchyma. The gallbladder neck can be easily freed from cystic plate and hepatic pedicle by pulling the gallbladder with appropriate force.

Since November 2022, we have performed this technique in total of 10 patients. The precise control of the ductal system allows for more accurate control of the hepatic parenchymal dissection process in the anatomical plane. Consequently, precise hepatectomy can be performed under better control, thereby further ensuring the safety of the operation. In this study, we aim to assess the benefits of integrating the cystic plate approach with real-time guided laparoscopic anatomical hepatectomy, in comparison with laparoscopic anatomical hepatectomy with conventional approach.

## Materials and methods

### Case selection

A total of 38 patients who met the inclusion criteria underwent laparoscopic hepatectomy were collected from January 2019 to November 2022. Ten cases were treated via cystic plate approach (marked as experimental group) and 28 cases were treated with conventional approach (marked as conventional group). All patients were postoperative pathologically confirmed primary liver cancer, there was no statistically significant difference in preoperative baseline data between two groups (Table 1).

**Table 1:** Preoperative clinical features between experimental and convention group.

Characteristics	experimental group	convention Group	*t*/χ^2^ value	*P*
Sex				
Male	8	25	0.556	0.456
Female	2	3		
HBsAg				
Positive	5	24	3.412	0.065
Negative	5	4		
AFP (ng/ml)				
≥400	0	8	2.104	0.147
<400	10	20		
Child-Pugh				
A	10	24	1.597	0.206
B	0	4		
Age (years)	62.6±8.1	57.1±8.8	1.743	0.090

According to the indications and contraindications of laparoscopic hepatectomy and ICG fluorescence display technique reported in the domestic and international literature, the inclusion criteria for laparoscopic hepatectomy were formulated in the context of this subject: a. Patients in good general condition without serious dysfunction of important organs such as heart, brain and lung; b. Well liver reserve function; c. No important vascular invasion and venous cancer thrombosis; and d. No distant tumor metastasis.

Exclusion criteria: a. Poor general condition of patients, unable to tolerate surgery or long period of pneumoperitoneum; b. Iodine allergy or ICG allergy; c. Moderate or severe impairment of liver reserve function; d. Large tumors that cannot be completely resected by laparoscopy; e. Tumors adjacent to the hepatic pedicle or invading large blood vessels; f. Preoperative imaging suggesting multiple intrahepatic metastases or distant metastases; g. Postoperative pathology confirming nonprimary liver cancer patients; and h. Previously undergone cholecystectomy.

Suitable patients were screened according to the inclusion criteria, and the liver reserve function was assessed preoperatively, and the liver volume, tumor location, size, adjacent vascular relationships, and the presence of bile duct vascular variants were accurately evaluated with the IQQA(R)-Liver Image Interpretation and Analysis System if necessary. All patients signed an informed consent form before surgery, which complied with medical ethics requirements. The work has been reported in line with the strengthening the reporting of cohort, cross-sectional, and case–control studies in surgery (STROCSS) criteria^5^ and the study was registered in Chinese Clinical Trial Registry (ChiCTR, UNI: 1800020329) https://www.chictr.org.cn/showproj.html?proj=31813

### Surgical resection

The surgical principle of this project: on the basis of not violating the existing principles of surgical resection of liver cancer, the surgical precut line was designed based on conventional preoperative and intraoperative multimodal imaging results, and the original resection line was modified according to the fluorescence of the liver surface and liver sections during hepatectomy. All fluorescence laparoscopic hepatectomies are performed using the conventional five-hole approach.

For routine removal of the gallbladder, the gallbladder bed can be freed along the gap between the Laennec capsule and the cystic plate, and the gallbladder can be completely stripped along with the cystic plate, at which point the right anterior hepatic pedicle can be revealed. (1) Dissection of the right hepatic pedicle: the square lobe of the liver is separated from the displaced part of the hilar plate (same as the left hepatic half), and the cystic plate is detached to clearly identify the right anterior hepatic pedicle to avoid misjudgment due to anatomical variation in the hilar region leading to vascular injury. (2) Dorsal dissection of the right hepatic tissues: dorsal dissection of the right hepatic tissues is also performed along the gap between Laennec’s capsule and Glisson’s sheath. The Glissonean pedicle of caudate process (G1c) can be dissociated first to fully open the dorsal space and facilitate dissection for freeing. (3) After adequate freeing, the right hepatic pedicle was ligated by using large right-angle forceps or ‘golden fingers’ to pass through the posterior tunnel of the hepatic pedicle and suspending the right hepatic pedicle with wires, and the right hepatic pedicle was completely clamped with pugilistic forceps, and the temporary blood flow control technique of Glisson’s pedicle was recommended for this step, that is, the right hepatic pedicle was temporarily clamped with pugilistic forceps, and the liver parenchyma was dissected first, and then the liver was completely split after the right hepatic pedicle dissected by applying a cutting closure. (4) After the liver parenchyma is sufficiently dissected, the right hepatic pedicle should be dissected with a cutting closure device on the right side of the ligation line or the right anterior and right posterior hepatic pedicle, respectively, to avoid biliary stenosis caused by dissection too close to the confluence of the left and right hepatic ducts.

In the conventional approach group, the first porta hepatis was blocked by the Pringle method, and the fibrous tissue connection between the porta hepatis and the Laennec membrane was bluntly detached from the ventral or dorsal side, respectively. This was followed by the lowering of the porta hepatis to reveal the root of the hepatic pedicle. The loose connective tissue between the porta hepatis and the Laennec membrane was then detached from the outside to the inside, thus revealing the pathway and branches of the target hepatic pedicle deep within the hepatic parenchyma. The target branches of the right hepatic pedicle were then identified, freed, and wrapped around. Once the target branch of the hepatic pedicle has been identified and freed, the dissection of the hepatic pedicle can be considered complete.

### ICG administration method

After exposing the Glisson system of the liver segment where the tumor is located, staining of the target liver segment is performed in two ways: positive staining and negative staining. Positive method: after locating the corresponding liver segment or subsegment portal vein under percutaneous or laparoscopic ultrasound guidance, or isolating the corresponding liver segment or subsegment Glisson, an appropriate amount of ICG (2.5 mg/ml) is injected into the portal vein of the corresponding liver segment or subsegment, and then the stained part of the liver is completely excised. Negative method: after separating the corresponding liver segment or subsegment Glisson, disconnect the corresponding Glisson system, inject 5 ml of ICG (0.5 mg/ml) through the peripheral vein, and then complete resection of the unstained part of the liver.

### Intraoperative detection

Real-time navigation using ICG fluorescence during resection of the liver parenchyma was used to continuously correct the plane of hepatectomy. If the target liver segment has thin portal vein branches or the target liver segment has difficulty in exposing the Glisson’s pedicle and the staining fails, the liver tumor border is explored by combining ICG fluorescence imaging technique with intraoperative ultrasound, and the liver incision margin is delineated at 1–2 cm from the tumor border, and the fluorescence mode is switched at any time during the liver incision to explore the tumor and ensure sufficient incision margin. All vessels and bile ducts encountered along the way should be separated in a reasonable way according to the diameter of the ducts. If intraoperative bleeding is uncontrollable, the hemorrhage should be promptly stopped by intermediate open surgery. If the liver section area is large, a small amount of diluted ICG (0.5 mg/ml) can be injected through the peripheral vein to assist in the detection and management of micro-biliary leaks by detecting the fluorescent signal in the liver section.

### Technical points and difficulties

(A) Preoperatively, clinicians should work together with imaging physicians to optimize scanning parameters and acquire high-quality CT image data to lay the foundation for establishing a three-dimensional visualization model of liver tumor; (B) Preoperatively and intraoperatively, the three-dimensional visualization model can be combined with all-round and multi-view dynamic observation of liver tumor and various vascular structures to guide the development of surgical plan; (C) When separating the cystic plate, the operator can along the ‘Red/Yellow Demarcation Line’ enter the correct Laennec extra-capsule gap, preset the hilar blocking band, and block the first hilar to reduce bleeding if necessary; small vessels can be ligated when descending the hilar; (D) ICG should be prepared with sterile injection water to avoid adverse reactions; (E) Depending on the positive or negative staining, choose different injection time and dose; (F) In patients with cirrhotic background, the fluorescence contrast between liver tumor tissue and the rest of liver tissue will be decreased, and the sensitivity of detection will be reduced.

### Statistical processing

SPSS 19.0 statistical software was used for statistical processing and analysis. *χ* ^*2*^ test was used for counting data, and the measurement data were expressed as mean±SD. *t*-test was used to compare the means of two samples, and paired *t*-test was used to compare before and after data of the same group. *P*<0.05 was the difference was statistically significant.

## Results

### Comparison of clinicopathological characteristics between the cystic approach group and conventional group

In our study, 10 patients were enrolled in the cystic approach group. All patients underwent fluorescence laparoscopic anatomical hepatectomy. Specifically, one patient underwent left hemi-hepatectomy, one underwent left inner lobe resection, one underwent middle lobe resection, three underwent right posterior lobe resection, and four underwent segment V/VI resection. Postoperative pathology confirmed the diagnosis of primary liver cancer in all cases, with negative resection margins. Two patients experienced postoperative complications, one with abdominal effusion and one with pleural effusion, both of whom recovered after treatment. The patients from conventional group were described previously^6^.

There was no significant difference in liver function, platelet and prothrombin time in the preoperative situation between the two groups (Table 2). The general condition and tumor size of total 38 patients were able to tolerate the surgery, the difference was not statistically significant. The cystic group did not demonstrate a significant prolongation of the operation time or an increase in blood loss (Table 3)^6^.

**Table 2:** Preoperative liver function index between experimental and convention group.

Characteristics	experimental group	convention group	*t*/χ^2^ value	*P*
ALT	33.5±18.9	39.4±36.6	−0.486	0.630
AST	31.6±14.6	39.7±49.9	−0.502	0.619
TB	14.8±2.8	14.9±5.3	−0.024	0.981
ALB	40.9±5.2	42.6±2.6	−1.316	0.197
PLT	164.6±44.2	133.1±52.4	1.631	0.112
PT	11.3±0.7	11.6±0.9	−0.932	0.357

**Table 3:** Intraoperative index between experimental and convention group.

Characteristics	experimental group	convention group	*t*/χ^2^ value	*P*
Tumor size (cm)	3.6±1.9	3.9±1.7	−0.592	0.557
Blood (ml)	240±129	322±236	−1.040	0.305
Operation time (min)	297±71	269±97	0.856	0.398

The postoperative indicators such as, the liver function indexes on the first postoperative day, WBC and the postoperative hospital stay, also had no statistical significance between the two groups (Table 4).

**Table 4:** Postoperative index between experimental and convention group.

Characteristics	experimental group	convention group	*t*/χ^2^ value	*P*
ALT	490±201	347±123	2.099	0.059
AST	513±204	371±104	2.096	0.061
TB	20.2±8.5	21.5±11.4	−0.320	0.751
ALB	34.5±3.6	35.9±3.1	−1.165	0.252
WBC	13.3±3.6	12.0±2.7	1.137	0.263
Hospitalization day	7.6±1.6	7.4±3.0	0.170	0.866

### The Red/Yellow demarcation line is a landmark for intervention of portal system

**Figure 1:**
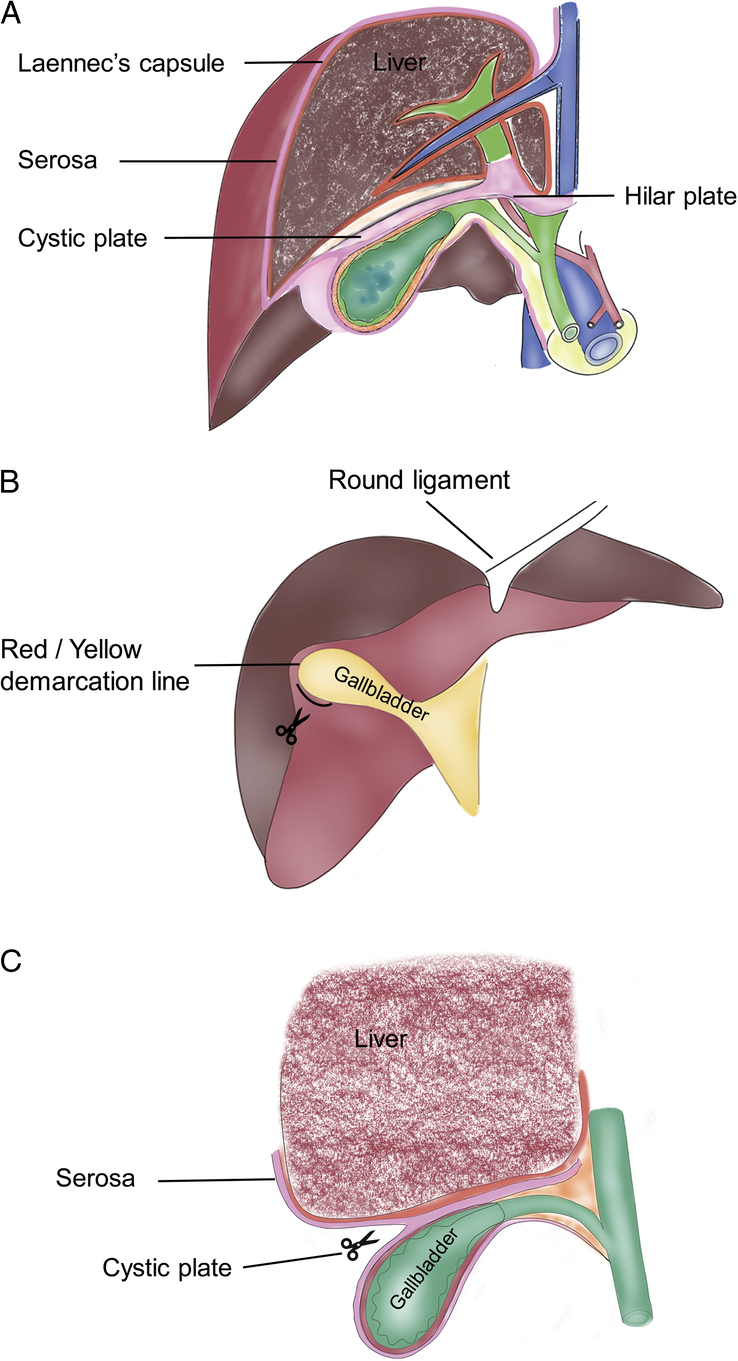
Schematic anatomy of cystic plate approach, (A) the location and essential components of cystic plate between liver and gallbladder; (B) the location of Red/Yellow Demarcation Line; (C) the illustration of cystic plate approach.

According to our study of hepatic membrane anatomy, we conclude that the cystic plate is composed of fused fascia, namely the hepatic lateral visceral peritoneum and the gallbladder lateral visceral peritoneum, as well as the gallbladder lateral sub-peritoneal fascia. There is a natural gap between the cystic plate and the hepatic lateral Laennec capsule. We introduced the concept of the ‘Red/Yellow demarcation line’ in the operation of the hilar system, which includes the descent of the cystic plate. The portal system and its accompanying adipose tissue have a yellow or yellow-white appearance compared to the red hue of the liver parenchyma (see Fig. 1A-C). The ‘Red/Yellow demarcation line’ indicates the peritoneal reflex line between the superficial plasma membrane of the portal system and the membrane that traverses the liver’s surface. This line is an important anatomical landmark in the operation of the hilar system, including the descent of the hilar plate and cystic plate.

**Figure 2:**
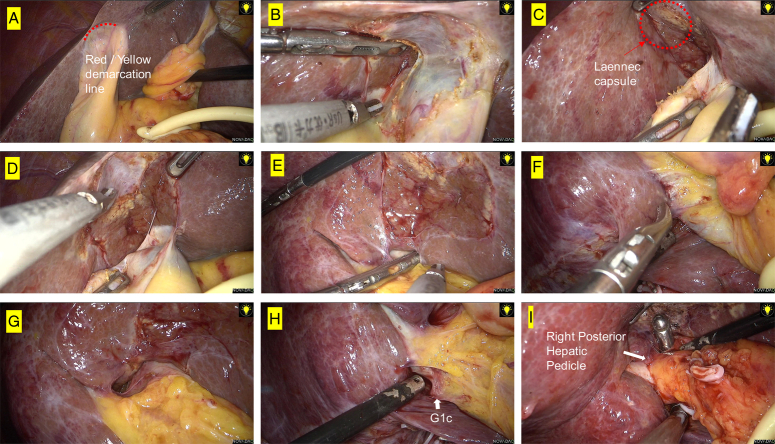
The cystic plate approach under laparoscopy, important structures were marked with red dashed circles and arrows. (A) Show the Red/Yellow demarcation line. (B) Enter the potential gap above the Red/Yellow demarcation line at the base of the gallbladder. (C) Hepatic Laennec capsule was show after cholecystectomy through cystic plate. (D–E) Complete removal of the gallbladder. (F–G) Separation the space between right anterior and right posterior hepatic pedicel. (H–I) Goldfinger complete free the right posterior hepatic pedicel.

To better understand the anatomical basis of the cystic plate approach via the Red/Yellow demarcation line, we used a case of laparoscopic anatomical hepatectomy as an example. The Red/Yellow demarcation line, as shown in Figure 2A, was identified first. To maintain tension between the gallbladder and the liver tissue migration, the base or body of the gallbladder was pulled (Fig. 2B), followed by entering the potential gap between the cystic plate and the hepatic Laennec capsule from just above the Red/Yellow demarcation line at the base of the gallbladder (Fig. 2C). The perforating vessels in the bed of the gallbladder were dissected using a combination of bipolar electrocoagulation and ultrasound knife coagulation. This was followed by a retrograde cholecystectomy with cystic plate (Fig. 2D–E). Blunt separation of hepatic tissues along the Glisson’s sheath allowed for exposure of the right half of the liver and the right anterior and right posterior Glisson’s sheath. The Glissonian branch for the caudate (G1c) and the right posterior lobe (right posterior hepatic pedicle) were exposed (Fig. 2G–I). Furthermore, the right anterior hepatic pedicle can also be easily dissected and separated (Fig. 2A–F) using the same cystic plate approach via the Red/Yellow demarcation line.

In summary, the cystic plate approach, which is based on the red-yellow demarcation line, provides a new, safe, simple, and effective method for separating the major Glissonian branches in the portal system. Here, we presented three typical cases in our study.

### The application of combining cystic plate approach and ICG fluorescence in laparoscopic liver lobectomy

Laparoscopic liver lobectomy has become a routine surgical method for the treatment of liver cancer in major central hospitals around the world. The advantages include lower complication rates, less blood loss, and shorter hospital stay in comparison to open lobectomy^7^. However, performing a laparoscopic lobectomy requires precise identification of the left or right hepatic pedicles and a clear ischemic boundary to guide manipulations on the liver parenchyma. Therefore, inexperienced surgeons, particularly beginners, and patients with abnormal anatomical structures may be at risk of unexpected blood loss and incorrect boundary recognition.

**Figure 3:**
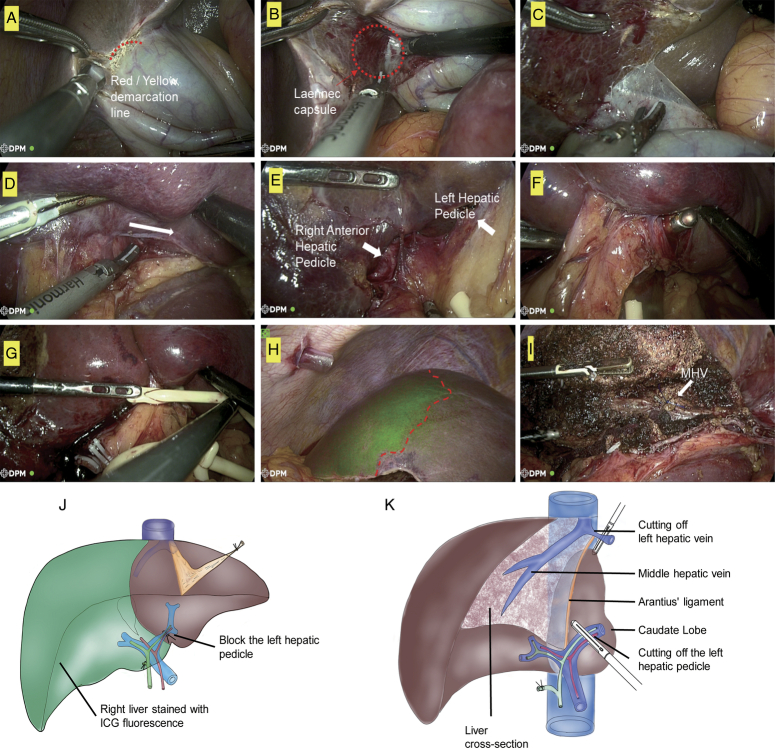
The application of cystic approach combining ICG fluorescence in the left hemi-hepatectomy. (A–D) The right hepatic pedicle was separated from Laennec capsule through Red/Yellow demarcation line. (E) Left hepatic pedicle and right pedicle were separated. (F) Goldfinger complete free the left hepatic pedicel. (G) The left hepatic pedicel was occluded. (H) 5ml of indocyanine green (0.5mg/ml) was injected intravenously for counterstain. (I) The left hemi-liver was safely and accurately separated along the boundary of the green stain. (J) Schematic showing ICG counterstaining after blocking the left hepatic pedicel. (K) Schematic showing fluorescence-guided left hemihepatectomy. ICG, indocyanine green; MHV, middle hepatic vein.

In this study, we introduced the application of ICG fluorescence combined with the cystic approach to facilitate safer and more precise liver lobectomy. As an example, we performed a left hemi-hepatectomy. After identifying the Red/Yellow demarcation line, we reached the right hepatic pedicle by bluntly separating the Laennec capsule (Fig. 3A–D). Subsequently, we exposed the left hepatic pedicle by bluntly separating it from the right pedicle all the way to the left (Fig. 3D and E). The left hepatic pedicle was isolated using the golden finger retractor and thoroughly occluded (Fig. F-G). Then, 5 ml of ICG (0.5 mg/ml) was injected intravenously. As a result, the right hemi-liver was marked with green stain, while the left part remained unaffected (Fig. H). Finally, the left hemi-liver was safely and accurately separated along the boundary of the green stain (Fig. I). The critical procedures were highlighted in the schematic diagrams (Fig. J-K).

### The application of combining cystic plate approach and ICG fluorescence in precise hepatic segmentectomy

Compared to hemi-hepatectomy, precise hepatic segmentectomy, particularly multiple segmentectomy, is more challenging. This is not only due to the complex vasculature of liver segments but also the depth at which these critical structures are located within the liver parenchyma. Furthermore, traditional laparoscopic hepatic segmentectomy has limitations in adequately exposing the target Glissonean branches and the adjacent ones that should remain untouched during the operation. However, the cystic approach can easily expose the Glissonean branches. Importantly, this approach creates a larger space for isolating and ligating the pedicles.

**Figure 4:**
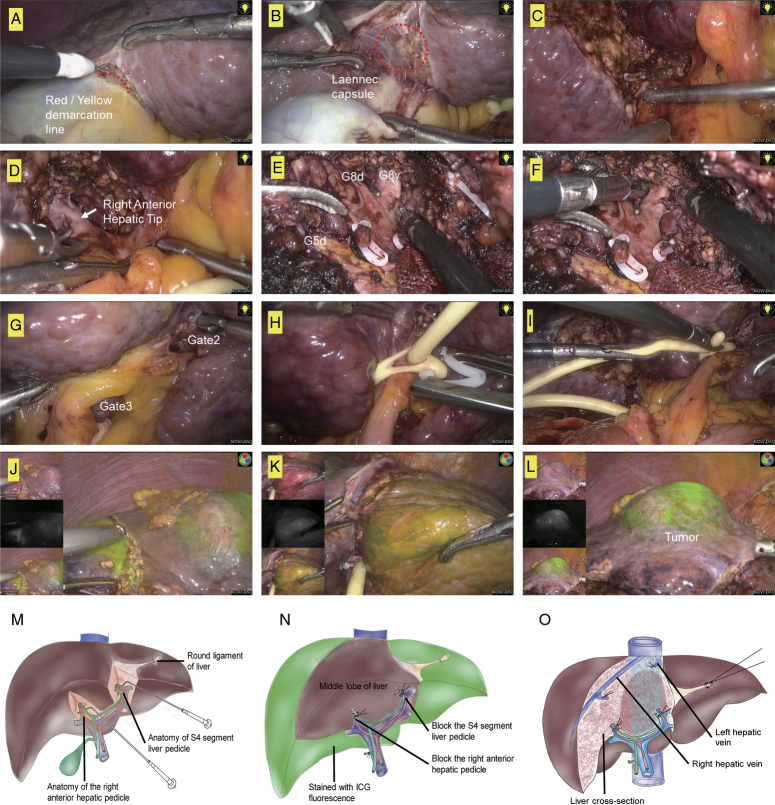
The application of combining cystic plate approach and ICG fluorescence in precise hepatic segmentectomy. (A–D) The right anterior hepatic pedicle was separated from Laennec capsule through Red/Yellow demarcation line. (E-F) Glissonean pedicle and subsequent right anterior branches (G5d, G8d and G8v) for hepatic segments 5 (S5) and 8 (S8) were separated. (G) Separate Gate II and III and isolated the Glissonean pedicle for S4. (H) Block Glissonean pedicle and subsequent right anterior branches for S5 and S8. (I) Block Glissonean pedicle for S4. (J–L) ICG counterstaining after blocking target-pedicel. DDG was performed three days before surgery to test liver reserve function, so the tumor was visualized. (M) Schematic showing blockage of the right anterior hepatic pedicle and S4 hepatic pedicle. (N) Schematic showing ICG counterstaining after blocking the target-hepatic pedicel. (O) Schematic showing fluorescence-guided middle hepatic lobectomy. ICG, indocyanine green.

In our study, we operated on a patient whose tumor was located at the junction of hepatic segments 4, 5, and 8 (G4, G5, and G8). In this case, we were able to locate the correct Glissonean pedicle and subsequent right anterior branches (G5d, G8d, and G8v) for hepatic segments 5 (G5) and 8 (G8) (Fig. 4A-F). Occlusion of these branches can affect the range of G5 and G8.

To identify G4, Sugioka *et al*. standardized the systematic isolation of the extrahepatic Glissonean pedicle. They found that connecting Gate II (the junction between the round ligament and the umbilical plate) and Gate III (the right edge of the Glissonean pedicle root of the umbilical portion) allows for isolation of the extrahepatic Glissonean pedicle of G4^3^. Therefore, we identified the Gate II and III and isolated the Glissonean pedicle for G4, followed by occluding the pedicle in order to identify the region of G4 (Fig. 4G-I).

In this case, combining the ICG fluorescence provides advantages in identifying the range of G4, G5, and G8. To ensure accurate assessment of liver reserve function before surgery, we perform DDG testing three days prior. During surgery, we use ICG staining to identify the tumor and gradually block the branches of the right anterior hepatic pedicle and the left intrahepatic lobe through a cystic plate approach. Additionally, we intravenously inject 5 ml of ICG (0.5 mg/ml) through a peripheral vein. The segment G1-3, G6, and G7 were shown under fluorescent laparoscopy with green stain, while the regions between the tumor (green) and undissected tissues had no stains (see Fig. 4J–L). Therefore, the G4, G5, and G8 can be clearly and safely dissected. The critical procedures were marked in Figure 4M–O.

### Cystic plate approach improves the precision in anatomical liver resection of HCC when orchestrating the ICG fluorescence

**Figure 5:**
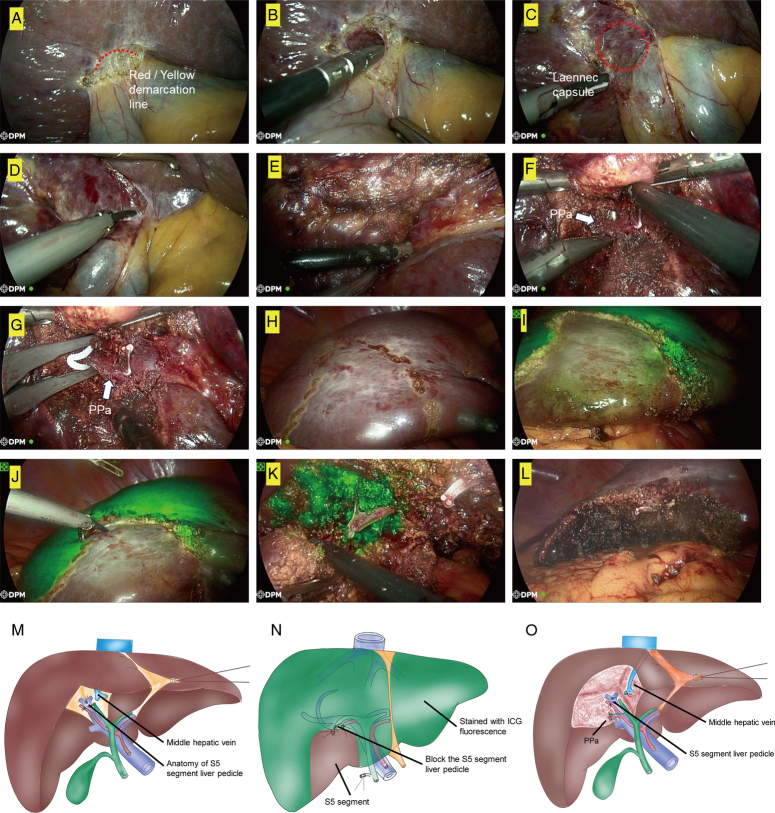
The application of combining cystic plate approach and ICG fluorescence in G5 sub-segmentectomy. (A–E) Complete removal of the gallbladder through Red/Yellow demarcation line. (F–G) PPa accurately isolated through extensive separation from the pedicle root. (H–I) ICG counterstaining marked a sub-segment of S5, and exhibited a distinct and well-defined boundary after PPa occluded. (J–L) Fluorescence-guided S5 resection. (M) Schematic showing Precision S5 excision. (N) Schematic showing ICG counterstaining after blocking PPa. (O) Schematic showing fluorescence-guided sub-segmentectomy. ICG, indocyanine green.

The study investigated the applications of ICG fluorescence in liver subsegment resection via cystic approach. For a patient with a tumor embedded at G5, we were able to limit the resection range by accurately isolating the first branch of the right posterior pedicle (PPa) through extensive separation from the pedicle root (see Fig. 5A–F, and M). This method is more efficient than the conventional way of splitting the liver. Additionally, it provides a clearer exposure of the branches of the posterior pedicle.

After blocking the PPa (as shown in Fig. 5G), we intravenously injected ICG to the patients. The counterstain marked a subsegment of liver G5, which is controlled by PPa, and exhibited a distinct and well-defined boundary (as shown in Fig. 5H and N). After dividing the liver along the PPa restriction and identifying the defined boundaries, the subsegment of G5 was safely removed (Fig. 5I–L, and O). It is important to note that the branches of Glissonean pedicles, which are not part of the PPa drainage basin, can be easily avoided, especially when operating in the depth of the liver parenchyma.

In summary, the cystic approach to controlling PPa has shown unprecedented convenience in precise liver resection for HCC when combined with ICG fluorescence.

## Discussion

Liver cancer is characterized by the propensity to metastasis along the hilar portal system. Anatomical hepatectomy, which is based on the physiological anatomy of the liver, has the capacity to completely remove micro-metastases within the hilar segment while simultaneously removing the main carcinoma. In contrast, nonanatomical hepatectomy may result in the retention of metastases within the hilar segment portal system, even if the margins are large enough, which can result in early recurrence of liver cancer after surgery^8^. The key technique of anatomic laparoscopic hepatectomy is to distinguish the complex anatomy of the liver, including the portal triad consisting of the hepatic artery, portal vein, and bile ducts, and its intricate relationship with the hepatic veins^9^. It is notable that almost all vascular and biliary variations occur within the hepatic portal system and its continuation in the intrahepatic Glisson’s sheath, which increases the difficulty and risk of hepatic resection^10^.

The Glisson sheath encases the hepatic hilar plate system, comprising the hilar plate, cystic plate, umbilical plate and the Arantian plate, along with the extrahepatic component, which is coated by the plasma membrane on the surface of the hepatoduodenal ligament^11^. The hepatic portal plate is a fibrous connective tissue that runs from above the confluence of the right and left hepatic ducts and into the surface of the hepatic vasculature. The intrahepatic Glisson’s sheath is the intrahepatic continuation of the hepatic portal plate system. The hepatic portal plate is connected to the cystic plate to the right, and the right hemihepatic Glisson’s pedicle is connected to the right end of the hepatic portal plate and extends intrahepatic. The right anterior Glisson’s pedicle is located externally and superiorly to the cystic plate, whereas the right posterior Glisson’s pedicle is located in the Rouviere’s groove just outside and below the cystic plate. To the left, it is connected to the umbilical plate, where the Glisson’s pedicle of hepatic segments II, III, and IV can be observed^12–14^.

The cystic plate approach becomes feasible due to the independence of the Laennec capsule with the Glisson sheath. However, its application in hepatectomy remains underreported, despite laparoscopic cholecystectomy through the cystic plate approach being deemed safe and feasible, with the additive benefit of avoiding the potential for intraoperative biliary tract injury. Based on the study of hepatic membrane anatomy, we were the first to propose the concept of the ‘Red/Yellow Demarcation Line’^15^, which is considered to be an important anatomical landmark for the manipulation of the hepatic portosystemic system (e.g. descending of the hepatic portal plate, gallbladder plate, etc.). The hepatic portal system and its adipose tissue are mostly yellow or yellowish-white, contrasting with the red color of the liver parenchyma, and the peritoneal reflex line between the superficial plasma membrane of the hepatic portal system and the peritoneal migration covering the plasma membrane of the hepatic surface is known as the Red/Yellow demarcation line. Unlike the conventional technique of descending the hepatic hilar plate directly at the hilum or separating the cystic plate for Glisson’s pedicle sheath dissection, in laparoscopic anatomical hepatectomy^16,17^, we operated on the basis of the ‘Red/Yellow demarcation line’, and entered the gallbladder from the bottom of the gallbladder just above the line. The incision was made from the bottom of the gallbladder, above the ‘Red/Yellow demarcation line’, into the potential gap between the cystic plate and the hepatic Laennec’s membrane. This was then followed to the neck of the gallbladder and the hepatic porta hepatis. Finally, the right hemihepatic and right anterior and posterior Glisson’s pedicles were identified, located in the deeper part of the cystic plate and connected to the hepatic portosystem. It was possible to dissect the hepatic pedicle of each segment of the right liver in the hepatic porta hepatis. Furthermore, the right liver could be dissected along the portosystem by separating to the left. The left half of the liver and the left external and left internal Glisson’s pedicle can also be dissected by separating along the hepatic portal plate system to the left side (further left hepatic pedicle can be dissected at the hilum), etc., we thus refer to this approach as the cystic plate approach based on the ‘Red/Yellow demarcation line’.

It is noteworthy that separation along the ‘Red/Yellow demarcation line’ typically allows access to the correct Glisson’s extrasheath or Laennec’s extramembranous space. However, deviation from this line frequently results in straightforward access to the intrathecal or intrahepatic area, which can potentially lead to hemorrhage due to injury of the intrathecal vasculature or vessels in the hepatic parenchyma. Once the ‘Red/Yellow Demarcation Line’ has been opened into the correct gap by blocking or not blocking the first hilar blood flow as appropriate, the separation can be attempted using a combination of blunt and sharp methods. When separating it to the first hilar, the gallbladder and the cystic plate can be pulled towards the left lower part of the liver with appropriate force, due to the continuity of the hepatic portosystemic plate with the Glisson’s sheath. This may assist in the exposure and separation of the Glisson’s pedicle. If the hepatic hilum is still too deep to be visualized intraoperatively, a small amount of surrounding hepatic tissue can be appropriately incised along the course of the pedicle, and then the target pedicle can be detached bluntly using a gold finger or other appropriate instrumentation. Then an appropriate amount of diluted ICG can be injected into the peripheral vein of the patient for ICG fluorescence counterstaining according to the patient’s body weight after clamping or blocking the Glisson pedicle of the corresponding hepatic regions or segments after separating the target hepatic pedicle. To note, the ICG fluorescence staining technique has been increasingly applied to laparoscopic anatomical hepatectomy, which can form a clear and long-lasting staining image among hepatic regions or segments, effectively displaying the liver and the liver. The technique can create a clear and persistent staining image of the hepatic region or intersegment, effectively display the most accurate intersegmental planes within the liver, and navigate the dissection of liver parenchyma by the operator in real-time^18–20^.

Therefore, we have optimized the two main and most time-consuming steps of anatomical hepatic resection, anatomical hepatic hilum and hepatic parenchyma resection, by combining the cholecystic plate approach based on the ‘Red/Yellow demarcation line’ with ICG fluorescence imaging. This has yielded favorable outcomes. All patients underwent successful surgery without intermediate openings, without Clavien–Dindo grade IIIa or higher complications, and without mortality.

In 2017, Sugioka *et al*.^3^ reported a technique for the separation of the hepatic Glisson’s pedicle through the gap between the hepatic portal system and Laennec’s membrane. Since then, some scholars in China have employed the hepatic portal separation technique to descend the hepatic portal and separate the hepatic pedicle of the right anterior lobe of the liver and the right posterior lobe of the liver outside of the Glisson’s sheath, resulting in enhanced outcomes^21^. The cystic plate approach, based on the ‘Red/Yellow demarcation line’, offers clear advantages over the technique of Sugioka *et al*., which emphasizes the ‘Red/Yellow Demarcation Line’ as the entry point of the hepatic portosystemic approach, such as the cystic plate. This approach allows the correct anatomical gap to be identified from the outset, facilitating the division of the hepatic pedicle into the right anterior lobe and the right posterior lobe of the liver outside the Glisson sheath. Furthermore, the significance of moderate gallbladder traction was underscored, which serves to facilitate the exposure and separation of the Glisson’s pedicle. This is achieved by appropriately tractioning the gallbladder and the cystic plate to the lower left side of the liver. This approach helps to prevent damage to the Glisson pedicle, while also separating the hepatic peritoneum (including Laennec’s membrane) from the liver parenchyma along with the cystic plate. In our opinion, if the potential gap between the cystic plate and the hepatic Laennec membrane is not accessed along the ‘Red/Yellow Demarcation Line’, the hepatic parenchyma can be damaged, resulting in increased intraoperative bleeding and a greater chance of tissue damage in the porta hepatis. In addition, Tokumitsu *et al*.^22^ chose to perform the cystic plate approach from Calot’s triangle rather than from the gallbladder floor during laparoscopic surgery, and this may be detrimental to the exposure and separation of the right hepatic Glisson’s pedicle due to the inability to obtain adequate traction.

To conclude, this study has the following advantages: (1) the separation along the ‘Red/Yellow demarcation line’ allows straightforward access to the correct Laennec’s membrane space, which is anatomically accurate; (2) The approach through the cystic plate is more convenient for the operator to expose and separate the Glisson’s pedicle by moderately tractioning the gallbladder and pulling the Glisson’s pedicle appropriately to the outside of the liver; (3) This approach has the same advantages as the traditional hepatic portal plate descent technique in that the Glisson’s pedicle can be separated outside the sheath, and it is less likely to damage the intrathecal vessels or the liver parenchyma, which is conducive to reducing intraoperative bleeding. (4) The use of ICG fluorescence real-time navigation ensures the accuracy of hepatic resection scope and shortens the operation time in some cases.

It is regrettable that the study was a single-center retrospective study with a limited number of patients, mainly with hepatocellular carcinoma, and no long-term follow-up results. Therefore, the technique, indications, contraindications and long-term oncologic efficacy of the extrasheath dissection of the hepatic tissues through the cystic plate approach need further confirmation.

In conclusion, the cystic plate approach based on the ‘Red/Yellow Demarcation Line’ combined with ICG fluorescence navigation technology can improve the safety and convenience of laparoscopic anatomical hepatectomy patients while realizing accurate hepatic resection, which has good clinical application value. The advancement of this technique suggests that it will be further promoted and utilized by an increased number of hepatobiliary surgeons, resulting in improved economic and social outcomes.

## Ethical approval

Ethical approval for this study (Ethical Committee N°2023-RE-213) was provided by the Ethical Committee NAC of The First Affiliated Hospital of USTC, HeFei, China on 29 June 2023.

## Consent

All patients signed an informed consent form before surgery.

## Source of funding

This study was supported by the Fundamental Research Funds for the Central Universities (No. WK9110000154), Tibet Natural Science Foundation (No. XZ2023ZR-ZY54(Z)), Anhui Provincial Natural Science Foundation (No. 2108085MH285).

## Author contribution

H.C., K.S., and X.S.: collected the related data and finished the manuscript and figures; P.Y.: did statistical analysis; Y.G., J.Y., W.L., and W.J.: did the operations; C.Z.: gave constructive guidance and made critical revisions; H.C. and P.Y.: participated in the design of this paper. All authors read and approved the final manuscript.

## Conflicts of interest disclosure

The authors have declared that no competing interest exists.

## Research registration unique identifying number (UIN)

All patients signed an informed consent form before surgery, which complied with medical ethics requirements (2023-RE-213); and registration number of clinical trial is ChiCTR1800020329.

## Guarantor

All authors read and approved the final manuscript.

## Data availability statement

All the data are available.

## Provenance and peer review

Not commissioned, externally peer-reviewed.
